# Tumor radioresistance caused by radiation-induced changes of stem-like cell content and sub-lethal damage repair capability

**DOI:** 10.1038/s41598-022-05172-4

**Published:** 2022-01-20

**Authors:** Roman Fukui, Ryo Saga, Yusuke Matsuya, Kazuo Tomita, Yoshikazu Kuwahara, Kentaro Ohuchi, Tomoaki Sato, Kazuhiko Okumura, Hiroyuki Date, Manabu Fukumoto, Yoichiro Hosokawa

**Affiliations:** 1 Department of Radiation Sciences, Graduate School of Health Science, Hirosaki Univesity, Hirosaki, Aomori 036-8564 Japan; 2 Nuclear Science and Engineering Center, Research Group for Radiation Transport Analysis, Japan Atomic Energy Agency (JAEA), Tokai, Ibaraki 319-1195 Japan; 3 Department of Applied Pharmacology, Kagoshima University Graduate School of Medical and Dental Sciences, Kagoshima University, Kagoshima, Kagoshima 890-8544 Japan; 4 Department of Radiation Biology and Medicine, Faculty of Medicine, Tohoku Medical and Pharmaceutical University, Sendai, Miyagi 983-8536 Japan; 5 Department of Oral and Maxillofacial Surgery, School of Dentistry, Health Sciences University of Hok-Kaido, Tobetsu-cho, Ishikari-gun, Hokkaido 061-0293 Japan; 6 Faculty of Health Sciences, Hokkaido University, Sapporo, Hokkaido 060-0812 Japan; 7 Pathology Informatics Team, RIKEN Center for Advanced Intelligence Project, Chuo-ku, Tokyo 103-0027 Japan

**Keywords:** Biophysics, Cancer

## Abstract

Cancer stem-like cells (CSCs) within solid tumors exhibit radioresistance, leading to recurrence and distant metastasis after radiotherapy. To experimentally study the characteristics of CSCs, radioresistant cell lines were successfully established using fractionated X-ray irradiation. The fundamental characteristics of CSCs in vitro have been previously reported; however, the relationship between CSC and acquired radioresistance remains uncertain. To efficiently study this relationship, we performed both in vitro experiments and theoretical analysis using a cell-killing model. Four types of human oral squamous carcinoma cell lines, non-radioresistant cell lines (SAS and HSC2), and radioresistant cell lines (SAS-R and HSC2-R), were used to measure the surviving fraction after single-dose irradiation, split-dose irradiation, and multi-fractionated irradiation. The SAS-R and HSC2-R cell lines were more positive for one of the CSC marker aldehyde dehydrogenase activity than the corresponding non-radioresistant cell lines. The theoretical model analysis showed that changes in both the experimental-based ALDH (+) fractions and DNA repair efficiency of ALDH (−) fractions (i.e., sub-lethal damage repair) are required to reproduce the measured cell survival data of non-radioresistant and radioresistant cell lines. These results suggest that the enhanced cell recovery in SAS-R and HSC2-R is important when predicting tumor control probability in radiotherapy to require a long dose-delivery time; in other words, intensity-modulated radiation therapy is ideal. This work provides a precise understanding of the mechanism of radioresistance, which is induced after irradiation of cancer cells.

## Introduction

Radiotherapy plays an important role in treating cancer and is often applied after surgical resection and current with chemotherapy, to achieve tumor control^1^. Recent radiation therapy uses modulated radiation intensity, so called intensity modulated radiation therapy (IMRT), which enables preserving organs at risk^2,3^. Also, stereotactic radiation therapy (SRT) gives high dose to solid tumors from many different angles^4,5^. Such advanced irradiation techniques require relatively longer dose-delivery times compared to previously used methods such as conformal therapy (3D-CRT)^2,6^, inducing cell recovery (radioresistance) by sub-lethal damage repair (SLDR)^7–9^. On the other hand, cancer stem-like cells (CSCs)^10^ with intrinsic or acquired radioresistance have higher DNA damage repair capability compared to non-CSCs^11^. Considering these biological characteristics, the possibility of recovery of CSCs between dose fractionations cannot be ignored. Therefore, the mechanisms underlying radioresistance in stem-like cells urgently need to be elucidated.

Radioresistant cell lines, such as oral squamous carcinoma SAS-R and HSC2-R, can be experimentally established after fractionated X-ray irradiations with 2 Gy/day for more than 30 days, which is often performed as a standard radiotherapy plan^12^. The CSC content can be evaluated using specific surface markers for CSCs and tumor formation ability^13^. Although fundamental biological data of radioresistant cell populations, including CSCs, have been experimentally accumulated^14,15^, the radioresistance mechanism remains uncertain because of the limited amount of experimental data. To solve this issue and to efficiently study the underlying mechanisms, we focused on a theoretical analysis using a biophysical model for predicting cell death. Among several biophysical models^16–21^, the integrated microdosimetric-kinetic (IMK) model has a unique feature that explicitly considers CSC content and DNA repair capability in heterogeneous cell populations^22–24^, enabling the theoretical evaluation of mechanisms underlying radioresistance and cell recovery of radioresistant cell lines.

In this study, we performed in vitro experiments and theoretical model analysis to investigate the radioresistance mechanisms of SAS-R and HSC2-R cell lines. This study provides a series of experimental survival assays of non-resistant cells (SAS, HSC2) and resistant cells (SAS-R, HSC2-R) after single-dose (acute) irradiation, split-dose irradiation and multi-fractionated irradiations, while a theoretical cell-killing model for reproducing the corresponding measured data proposes a potential scenario of radioresistance development after fractionated irradiation. In this study, we discuss the tumor radioresistance mechanisms and damage recovery of radioresistant cells during irradiation. This study based on cell experiments and theoretical estimation of cell-killing would provide precise understanding of mechanisms contributing to tumor radioresistance during fractionated radiotherapy.

## Materials and methods

### Cell culture

In this study, we chose the human oral squamous carcinoma cell lines SAS and HSC2, and their radioresistant counterparts SAS-R and HSC2-R as models^12^. These cell lines were obtained from the Cell Resource Center for Biomedical Research, Institute of Development, Aging and Cancer, Tohoku University. It should be noted that SAS-R and HSC2-R acquired radioresistance after exposed to daily 2 Gy of X-rays for more than 1 year^12^. These cell lines were maintained at 37 ℃ and 5% CO_2_ environment in Roswell Park Memorial Institute 1640 medium (Thermo Fisher Scientific, Inc. Tokyo, Japan) supplemented with 10% heat-inactivated fetal bovine serum (FBS) (Japan Bio Serum, Fukuyama, Japan) and 1% penicillin/streptomycin (Life Technologies, CA, USA).

### Irradiation conditions

In single dose experiment, the cultured cells were irradiated with kilo-voltage X-rays (150 kVp, 1.0 Gy/min) through an additional filter 0.5 mm aluminum and 0.3 mm copper using an X-ray generator (MBR-1520R-3; Hitachi Medical Co., Ltd., Tokyo, Japan). Split-dose experiment was performed with 4 Gy in two divided doses (i.e., 2 Gy irradiation twice) at various inter-fraction times. The same X-ray generator as the single dose experiment was used in case of split-dose experiment. To achieve various dose rates, 0.1 and 0.25 Gy/min, we performed multi fractionation experiment. For 0.1 Gy/min, 6 or 10 Gy irradiation was fractionated and irradiation time was 60 and 100 min, respectively. For 0.25 Gy/min, irradiation time was 24 and 40 min. The fractionation size was 1 Gy (1.0 Gy/min). The dose-averaged linear energy transfer (LET_D_) and the dose-mean lineal energy (*y*_*D*_) were estimated to be 1.53 kV/µm and 4.68 keV/μm, respectively, using the Particle and Heavy Ion Transport code System (PHITS) ver. 3.21^25^, and the in-house code of WLTrack for electrons^26^. The dose in air was monitored with a thimble ionization chamber placed next to the sample during irradiation. The uncertainty of the absorbed dose measured by the thimble ionization chamber was ± 1%.

### Colony formation assay

Clonogenic potency was evaluated using a colony formation assay. The appropriate number of cells were seeded on φ60 or φ100 cell culture dishes and irradiated with X-rays after 2 h incubation to allow cells to adhere to the bottom of the dish. The cells were fixed with methanol (Wako Pure Chemical Industries, Ltd., Osaka, Japan) 7–10 days after irradiation, and stained with Giemsa staining solution (Wako Pure Chemical Industries). Colonies with more than 50 cells were counted. The surviving fraction for each cell line was calculated from the ratio of the plating efficiency of irradiated cells to that of non-irradiated cells.

### Flow cytometric analysis for detecting the ALDH positive fractions

To input the reference value of CSC fraction into the theoretical model, we selected ALDH which is reported as CSC marker for head and neck cancer^27^, and measured the CSC fraction for each cell line. The ALDEFLUOR Kit (Catalog no. 01700) was purchased from STEMCELL Technologies, Inc. (Vancouver, Canada). Trypsinized cells were adjusted to a density of 1 × 10^6^ cells/mL and washed twice with phosphate-buffered saline. Next, the cells were incubated for 45 min at 37 ℃ in the dark after the addition of ALDEFLUOR reagent (5 μL/10^6^ cells). For the negative control, 1.5 mM diethylaminobenzaldehyde (DEAB) was added at a final concentration of 7.5 μM. Subsequently, the cells were centrifuged, resuspended in ALDEFLUOR assay buffer, and analyzed by direct immunofluorescence flowcytometry using a FACS Aria (BD Biosciences, Tokyo, Japan). The percentage of ALDH (+) was determined by subtract from the positive percentage of DEAB administration group with about 0.5% false positives. Noted that the ALDH (+) cell populations was assumed to correspond to a radioresistant population. In SAS-R and HSC2-R cells, the ALDH activity within six months (i.e., passage number of 20–50) after the last irradiation.

The experiment was repeated three times. The significances of differences between non-irradiated group and irradiated group were evaluated using Student’s t-test. *P* < 0.01 was considered to indicate a statistically significant difference, which is noted as ** in this study.

### A theoretical cell-killing model for analyzing measured survival data

To theoretically analyze the cell survival data measured by clonogenic survival assay, we employed a theoretical cell-killing model, the “IMK model”, which considers microdosimetric quantities along ionizing radiation tracks, cell recovery during irradiation, and the existence of stem-like cells^22–24^. In this section, we provide an overview of the IMK model used in this study.

### Surviving fraction after single-dose irradiation for a certain single cell population

In the IMK model, it is assumed that the nucleus is radiation sensitive target divided into hundreds of small sites (called domains) with a diameter of 1–2 μm. By considering the energy deposited in every domain and repair kinetics of induced DNA lesions in a domain, the model enables the evaluation of the impact of microdosimetry and SLDR on cellular damage after irradiation^23,24^. To consider the repair kinetics of DNA lesions during and after irradiation, the model incorporates potentially lethal lesions (PLLs) that can undergo one of three transformations: (i) a PLL can transform into a lethal lesion at a constant rate *a* (h^−1^); (ii) two PLLs can transform into a LL at a constant rate *b*_d_ (h^−1^); (iii) a PLL can be repaired by DNA damage repair pathway (mainly non-homologous end joining (NHEJ)) at a constant rate *c* (h^−1^)^28^. By solving the kinetic equations of PLLs, the surviving fraction for various irradiation conditions (i.e., single-dose irradiation, split dose irradiation, and multi-fractionated irradiations) can be expressed based on the linear-quadratic relation.

First, we assume that a single dose is delivered to a single-cell population with the dose-delivery time *T* (h). This modeling was discussed in our previous report on the IMK model^23^. Thus, the surviving fraction of a certain single cell population *S* after single-dose irradiation can be expressed as:

1 $$ - \ln S = \left( {\alpha_{0} + \frac{{y_{D} }}{{\rho \pi r_{d}^{2} }}\beta_{0} } \right)D + F\beta_{0} D^{2} , $$

where *D* is the absorbed dose (= $\dot{D}$ *T*); $\dot{D}$ is the absorbed dose rate (Gy/h); *y*_D_ is dose-mean lineal energy (keV/μm) representing the radiation track structure^29^; *ρ* and *r*_d_ represent the density of liquid water (1.0 g/cm^3^) and radius of a domain (set as 0.5 μm in this study), respectively; *F* is the Lea-Catcheside time factor, which is expressed as

2 $$ F = \frac{2}{{(a + c)^{2} T^{2} }}\left[ {(a + c)T + e^{ - (a + c)T} - 1} \right]. $$

Note that (*a* + *c*) can be approximated as *c*, representing the SLDR rate (h^−1^)^23^. $\alpha_{0}$ and $\beta_{0}$ are the coefficients for the dose (Gy^−1^) and dose squared (Gy^−2^), respectively, which are in inversely proportional to (*a* + *c*)^23^ as

3 $$ \alpha_{0} \propto \frac{1}{(a + c)} \cong \frac{1}{c}\;{\text{and}}\;\beta_{0} \propto \frac{1}{(a + c)} \cong \frac{1}{c}. $$

Using Eqs. (1)–(3), we can estimate tumor cell survival for various dose rates based on the cell-specific parameters of a certain single cell population [$\alpha_{0} , \beta_{0} ,$ (*a* + *c*)].

### Surviving fraction after split-dose irradiation for a certain single cell population

Next, we assume that a certain single cell population is exposed to two irradiation doses with inter-fraction time $\tau$ (h). Based on previous reports^23,30^, the surviving fraction for split-dose irradiation can be expressed as:

4 $$ - \ln S(\tau ) = \mathop \sum \limits_{i = 1}^{2} \left[ {\left( {\alpha_{0} + \frac{{y_{D} }}{{\rho \pi r_{d}^{2} }}\beta_{0} } \right)D_{i} + \beta_{0} D_{i}^{2} } \right] + 2\beta_{0} e^{ - (a + c)\tau } D_{1} D_{2} . $$

Note that we assume that two doses (*D*_1_ and *D*_2_) are acutely delivered to the cell population. By using the surviving fractions taking the limits of the fractionation time ($\tau \to 0, \tau \to \infty$), *S*(0), and *S*($\infty$), the SLDR rate can be calculated from the experimental split-dose cell recovery curve. The SLDR rate can be expressed based on the previous modeling^23^ as

5 $$ (a + c) = \frac{{\mathop {\lim }\limits_{\tau \to 0} \frac{1}{S}\frac{{{\text{d}}S}}{{{\text{d}}\tau }}}}{{\ln \frac{S(\infty )}{{S(0)}}}}. $$

As perprevious reports, the mean value of (*a* + *c*) for cancer cell lines ranges from 1.506 to 2.218 (h^−1^)^23^.

### Surviving fraction considering progeny cells and cancer stem-like cells

**Figure 1:**
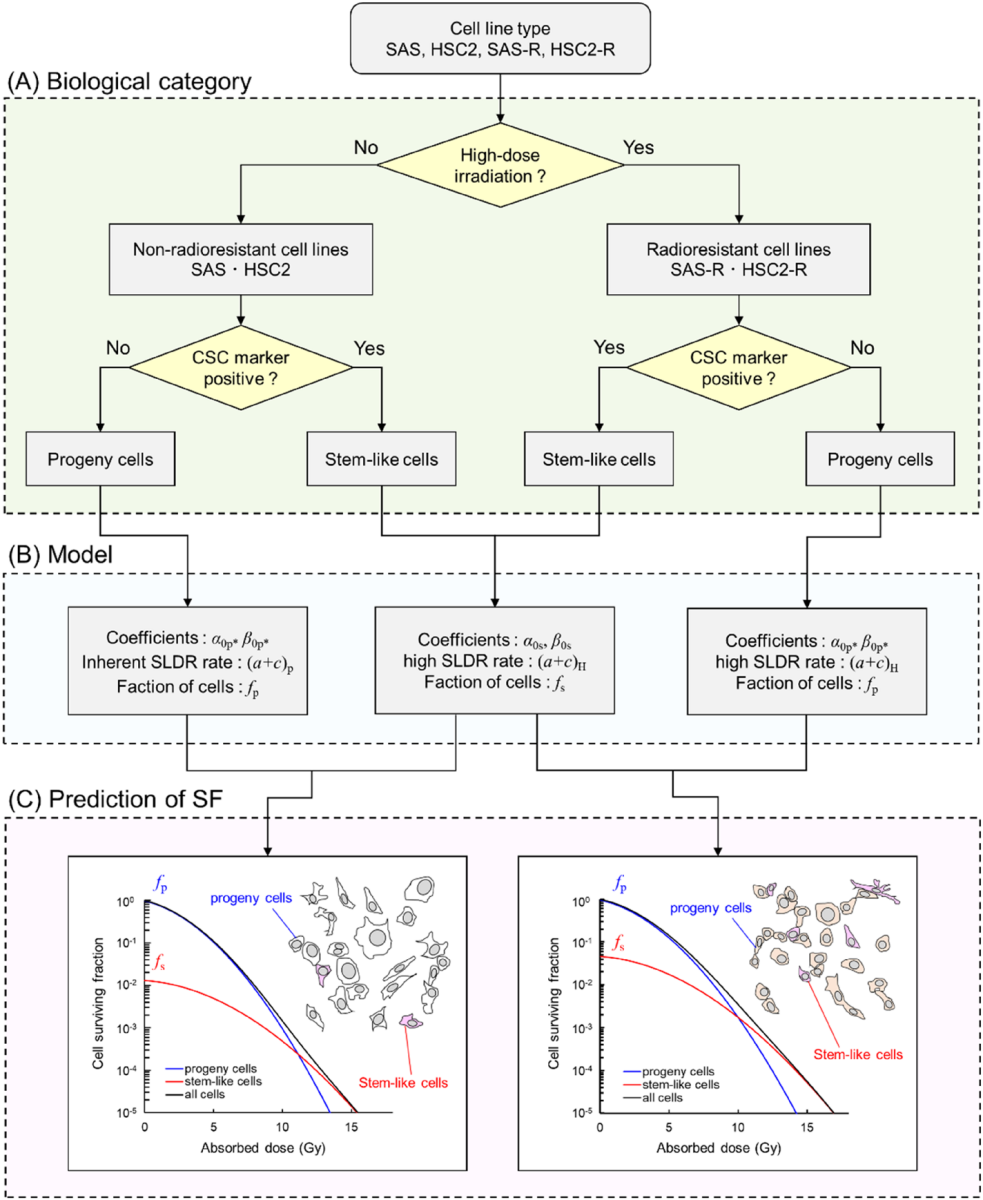
Categories of cancer cell lines and the overview of the IMK model. (A) the biological categories for SAS, SAS-R, HSC2 and HSC2-R, (B) the parameters in the IMK model and their characteristics, and (C) surviving fraction of cancer cells estimated based on the IMK model. Note that *f*_p_ + *f*_s_ = 1. We assumed the increased fraction of CSCs and the high DNA repair (SLDR) capability acquired in the progeny cells after fractionated irradiation with total high dose as the characteristics of radioresistant cell lines.

Cells that survive fractionated irradiation can acquire a greater radioresistant ability than the non-irradiated (non-radioresistant) parental cells, as shown in Fig. 1A. Experiments suggest that the radioresistant cell line exhibits enhanced DNA repair capability and a higher CSC fraction compared to non-radioresistant cell lines^14,31^. To account for these cellular characteristics, we introduced a two-cell population model^22^ and the enhancement factor of SLDR, *w*_SLDR_. Using the model for a single cell population (i.e., Eqs. (1) and (2)), the surviving fractions for progeny cells and CSCs can be expressed as

6 $$ - \ln S_{{\text{p}}} = \left( {\alpha_{{0{\text{p}}*}} + \frac{{y_{D} }}{{\rho \pi r_{{\text{d}}}^{2} }}\beta_{{0{\text{p}}*}} } \right)D + \frac{{2\beta_{{0{\text{p}}*}} }}{{(a + c)_{{{\text{p}}*}}^{2} T^{2} }}\left[ {(a + c)_{{{\text{p}}*}} T + e^{{ - (a + c)_{{{\text{p}}*}} T}} - 1} \right]D^{2} $$

7 $$ - \ln S_{{\text{s}}} = \left( {\alpha_{{0{\text{S}}}} + \frac{{y_{D} }}{{\rho \pi r_{{\text{d}}}^{2} }}\beta_{{0{\text{s}}}} } \right)D + \frac{{2\beta_{{0{\text{s}}}} }}{{(a + c)_{{\text{H}}}^{2} T^{2} }}\left[ {(a + c)_{{\text{H}}} T + e^{{ - (a + c)_{{\text{H}}} T}} - 1} \right]D^{2} $$

where *S*_p_ and *S*_s_ are the surviving fractions for progeny cells and CSCs, respectively; [$\alpha_{{0{\text{p}}*}} , \beta_{{0{\text{p}}*}} ,$ (*a* + *c*)_p*_], and [$\alpha_{{0{\text{s}}}} ,\beta_{{0{\text{s}}}} ,$ (*a* + *c*)_H_] are model parameters for progeny cells and CSCs, respectively; (*a* + *c*)_p*_ is the SLDR rate of progeny cells, and (*a* + *c*)_H_ is the SLDR rate of CSCs. Based on the experimental report^15^, we assumed that the DNA repair efficiency (SLDR rate) of progeny cells in the radioresistant cell populations was enhanced compared to that of the non-radioresistant parental cells. With this assumption, we can express that the set of parameters for progeny cells can be modulated by the (*a* + *c*) value. The parameter set of [$\alpha_{{0{\text{p}}*}} , \beta_{{0{\text{p}}*}} ,$ (*a* + *c*)_p*_] can be expressed as follows

8 $$ \begin{aligned} \left( {a + c} \right)_{{{\text{p}}*}} & = \left( {a + c} \right)_{{\text{p}}} \left[ {{\text{for}}\;{\text{non-radioresistant}}\;{\text{cells}}} \right] \\ & = (a + c)_{{\text{H}}} \left[ {{\text{for}}\;{\text{radioresistant}}\;{\text{cells}}} \right] \\ \end{aligned} $$

9 $$ w_{{{\text{SLDR}}}} = \frac{{(a + c)_{{\text{H}}} }}{{(a + c)_{{\text{p}}} }} $$

10 $$ \alpha_{{0{\text{p}}*}} = \frac{{\alpha_{{0{\text{p}}}} }}{{w_{{{\text{SLDR}}}} }} $$

11 $$ \beta_{{0{\text{p}}*}} = \frac{{\beta_{{0{\text{p}}}} }}{{w_{{{\text{SLDR}}}} }}, $$

where (*a* + *c*)_p_ is the inherent SLDR rate in non-radioresistant parent (progeny) cells. The model parameters for progeny cells, progeny cells with increased SLDR rates, and CSCs are summarized in Fig. 1B.

Similar to single-dose irradiation, the surviving fractions of progeny cells and CSCs in case of split-dose irradiation can be expressed as

12 $$ - \ln S_{{\text{p}}} (\tau ) = \mathop \sum \limits_{i = 1}^{2} \left[ {\left( {\alpha_{{0{\text{p}}*}} + \frac{{y_{D} }}{{\rho \pi r_{{\text{d}}}^{2} }}\beta_{{0{\text{p}}*}} } \right)D_{i} + \beta_{{0{\text{p}}*}} D_{i}^{2} } \right] + 2\beta_{{0{\text{p}}*}} e^{{ - (a + c)_{{{\text{p}}*}} \tau }} D_{1} D_{2} $$

13 $$ - \ln S_{{\text{s}}} (\tau ) = \mathop \sum \limits_{i = 1}^{2} \left[ {\left( {\alpha_{{0{\text{s}}}} + \frac{{y_{D} }}{{\rho \pi r_{{\text{d}}}^{2} }}\beta_{{0{\text{s}}}} } \right)D_{i} + \beta_{{0{\text{s}}}} D_{i}^{2} } \right] + 2\beta_{{0{\text{s}}}} e^{{ - (a + c)_{{\text{H}}} \tau }} D_{1} D_{2} $$

As shown in Fig. 1, we considered that the cancer cell line is composed of two cell populations: progeny cells and CSCs. By considering the surviving fraction for the cell population containing the progeny cells and CSCs, the overall surviving fraction *S* can be expressed as

14 $$ S = S_{{\text{p}}} f_{{\text{p}}} + S_{{\text{s}}} f_{{\text{s}}} $$

where $f_{{\text{p}}}$ is the fraction of progeny cells, and $f_{{\text{s}}}$ is the fraction of CSCs. Note that *f*_p_ + *f*_s_ = 1. As shown in Fig. 1B, the differences in cell-specific parameters between non-radioresistant parental cell lines and radioresistant cell lines are (i) an increase in the SLDR rate in progeny cells and (ii) fraction of CSCs. As shown in Fig. 1C, using Eqs. (6)–(12) and (15), we analyzed the surviving fractions of non-radioresistant cell lines (SAS and HSC2) and radioresistant cell lines (SAS-R and HSC2-R) measured in this study.

### Determination of the model parameters

The model parameters were determined by applying the present model to the experimental survival data after single-dose and split-dose irradiation. The steps to obtain the model parameters are: (i) determination of the (*a* + *c*) values using the experimental split-dose cell recovery curve and Eq. (5), and (ii) determination of the cell-specific parameters [*α*_0p_, *β*_0p_, (*a* + *c*)_p_, *α*_0S_, *β*_0S_, *w*_SLDR_] by applying Eqs. (6)–(12) and (15) to the experimental surviving fraction after single-dose irradiation of both non-radioresistant and radioresistant cell lines. For the latter determination, we used the Markov chain Monte Carlo (MCMC) simulation^32,33^. The algorithms of MCMC are summarized in previous reports^23,34^, which enable the evaluation of the uncertainties of model parameters.

In the MCMC simulation, the prior distributions of *α*_0p_, *β*_0p_, *α*_0S_, *β*_0S_, and *w*_SLDR_ were set to be uniform. We also assumed that the parameters of stem-like cells [$\alpha_{{0{\text{s}}}} ,\beta_{{0{\text{s}}}}$] are smaller than those of progeny cells [$\alpha_{{0{\text{p}}}} ,\beta_{{0{\text{p}}}}$] based on our previous study^22^. The prior distribution of (*a* + *c*)_p_ was obtained from the model analysis using the experimental split-dose cell recovery curve of the non-radioresistant cell lines (SAS, HSC2), while that of *f*_s_ was the experimental flowcytometric data itself. The set of model parameters *θ*[*α*_0p_, *β*_0p_, (*a* + *c*)_p_, *α*_0S_, *β*_0S_, *w*_SLDR_, *f*_p_, *f*_s_] was sampled following the likelihood *P*(*d*_*i*_|*θ*) and the transition probability *α*_P_ as follows:

15 $$ P(d|\theta ) = \mathop \prod \limits_{i = 1}^{N} [P(d_{i} |\theta )] = \mathop \prod \limits_{i = 1}^{N} \left\{ {\frac{1}{{\sqrt {2\pi \sigma } }}\exp \left[ { - \frac{{\left( { - \ln S_{\exp i } + \ln S_{{{\text{cal}}i}} } \right)^{2} }}{{2\sigma^{2} }}} \right]} \right\} $$

16 $$ \alpha_{{\text{P}}} = \frac{{P\left( {\theta^{{{\text{candidate}}}} | d} \right)}}{{P\left( {\theta^{(t)} |d} \right)}} $$

where *d*_*i*_ (*i* = 1 ~ *N*) is the experimental survival data [*d*_*i*_ = (*D*_*i*_, − ln *S*_exp*i*_)], *S*_exp_ is the experimental surviving fraction measured by the colony formation assay, *S*_cal_ is the surviving fraction calculated by the model, and *P*(*θ*|*d*) and *P*(*θ*^*candidate*^|*d*) are the posterior likelihood for the candidate (*t* + 1)-th and the previous (*t*)-th conditions, respectively, as reported previously^24^.

After sampling the set of parameters via MCMC, we calculated the mean values and standard deviations of the model parameters. Note that the parameters *f*_S_ and (*a* + *c*)_p_ were updated using the MCMC simulation. Using the mean values, we calculated the surviving fraction to analyze the radiosensitivity of non-radioresistant and radioresistant cell lines. To check whether the present IMK model is in agreement with the experimental data, we calculated the coefficient of determination (*R*^2^) value.

## Results

### Determination of SLDR rate from split-dose irradiation experiment

**Figure 2:**
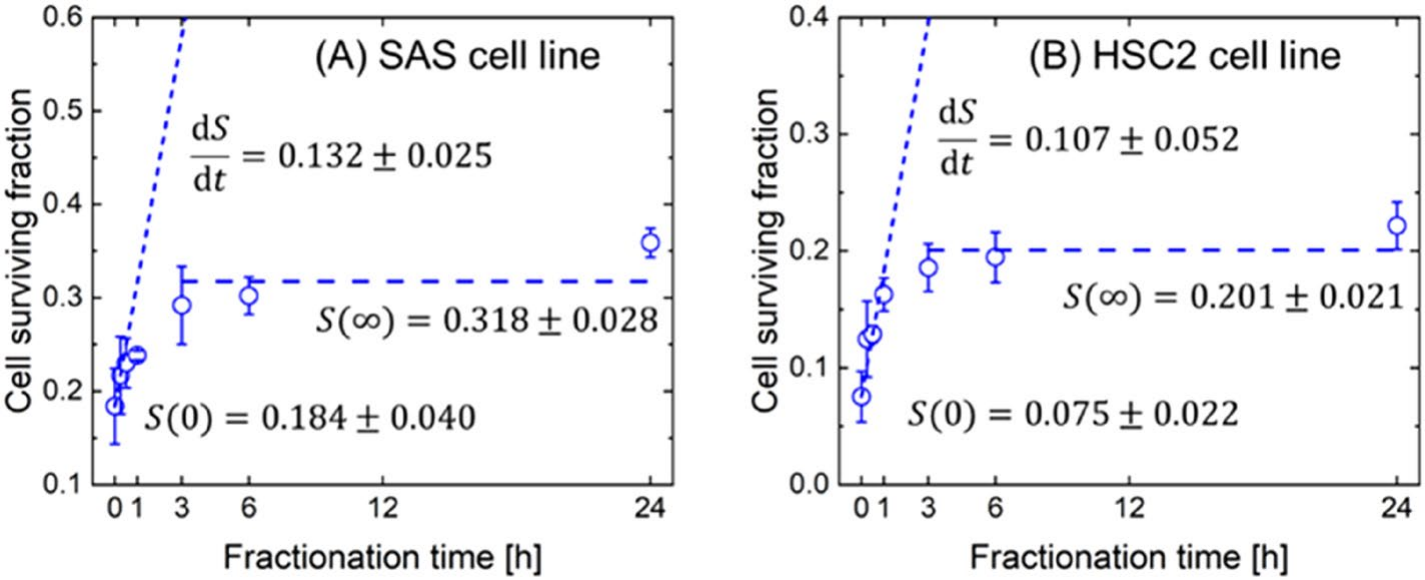
Determination of (*a* + *c*) values for non-resistant cell lines. (**A**) is cell recovery curve of SAS and (**B**) is that of HSC2. The symbols are shown the experimental cell surviving fraction of fractionated radiations as a function of time intervals (h), while two dotted lines are initial slopes of d*S*/d*τ* and *S*(∞) expressed in Eq. (5), respectively. Using the values described in this figure, the SLDR rates of non-radioresisitant cell lines (SAS, HSC2) were determined.

To obtain the prior information on the SLDR rate (i.e., *c* in h^−1^), we measured the surviving fraction after split-dose irradiation and applied Eq. (5) to the measured survival data of non-radioresistant SAS and HSC2 cell lines. Figure 2 shows the split-dose cell recovery curve, where (A) is the curve of SAS and (B) is that of HSC2. The open circles represent the experimental results, while the two dotted lines indicate the initial slopes of d*S*/d*τ* and *S*(∞) expressed in Eq. (5), respectively. As shown in Fig. 2, the cell surviving fraction of both cell lines increased monotonically in the interval range of 0–3 h, and the recovery was saturated in the interval range of 6–24 h. Cell recovery between dose fractionation reflects the SLDR of cell survival^23^.

By using the values of d*S*/d*τ, S*(∞) and *S*(0) described in Fig. 2 and Eq. (5), the SLDR rates ((*a* + *c*) value) of SAS and HSC2 were estimated to be 1.31 ± 0.69 (h^−1^) and 1.45 ± 0.93 (h^−1^), respectively. Even in the case of the same oral squamous carcinoma cell line, the SLDR rate of HSC2 was slightly higher than that of SAS. The previous MK model analysis suggested that PLLs correspond to DNA double-strand breaks^35^. It is well known that two major repair pathways, NHEJ and homologous recombination (HR), can repair radiation-induced DNA double strand-breaks (DSBs)^36^, but the SLDR rate mainly comprises the faster component of NHEJ^37^. The difference between SAS and HSC might be attributed to the cell-cycle distributions. However, judging from their uncertainties, the results suggest that the (*a* + *c*) values of SAS and HSC2 are similar.

### Measurement of the ALDH-positive fractions by flowcytometric analysis

**Figure 3:**
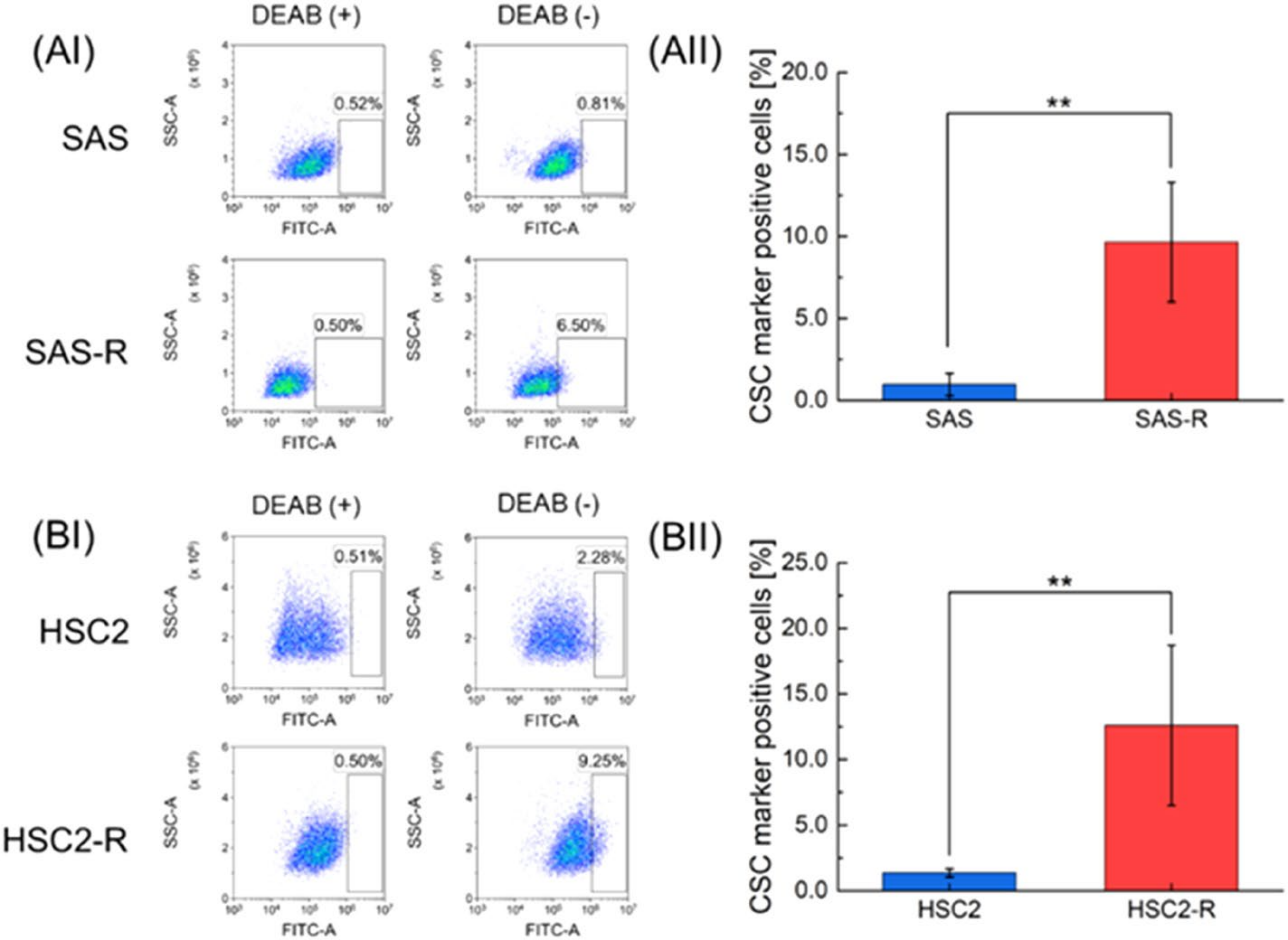
The ALDH (+) population in four cell lines. The fraction of cells expressing ALDH activity was determined by flowcytometric analysis. The cells that tested positive for ALDH activity are shown in the scatter plot on the right. Representative cytogram of (AI) SAS and SAS-R cells, (BI) HSC2 cells and HSC2-R cells. DEAB (+) represents the negative control. (AII) and (BII) show the percentages of ALDH (+) cells subtracted from that of DEAB (+). Bracketed asterisks represent significant differences of *P* < 0.01 between the two groups.

The IMK model includes several model parameters. To efficiently determine the parameter sets, we experimentally determined the ALDH (+) fractions (assuming *f*_s_ value) in the cell lines used in this study. In general, the abundance of ALDH (+) in the cell population can be measured by flowcytometric analysis. In this study, ALDH was used to determine the radioresistant population. Figure 3 shows the experimental results of the ALDH (+) fraction of each cell line: (A) SAS and SAS-R, and (B) HSC2 and HSC2-R. In Fig. 3, the percentages of ALDH (+) cells were 0.97 ± 0.68% for SAS, 9.65 ± 3.65% for SAS-R, 1.36 ± 0.32% for HSC2, and 12.61 ± 6.11% for HSC2-R. From these measured ALDH (+) fractions, it was confirmed that the SAS-R and the HSC2-R included more stem-like cells than SAS and HSC2, which suggests that the radioresistance acquired after fractionated irradiation is intrinsically related to the increment of ALDH (+) fractions.

### Dose–response curve fitting by theoretical model

**Figure 4:**
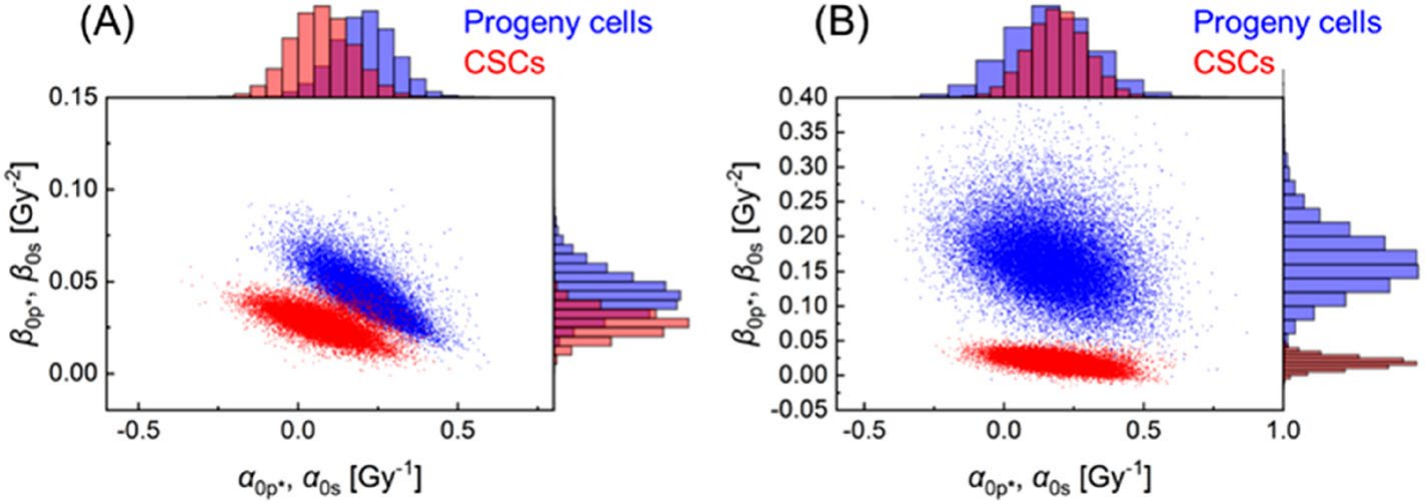
Model parameter sets of non-radioresistant cell lines by the MCMC simulations. The dot plot and probability density histogram represent the posterior distributions of the model parameter sets of (**A**) SAS, and (**B**) HSC2. Blue represents the parameters of [*α*_0p*_, *β*_0p*_] for the progeny cells in the non-resistant cell populations, red represents the parameters of [*α*_0s_, *β*_0s_] for the CSCs in the non-resistant cell populations.

Using the experimental values of (*a* + *c*) and *f*_s_ as prior information, we analyzed the dose–response curve of the cell surviving fraction. We performed the MCMC simulation to determine the parameter sets (*α*_0p*_, *β*_0p*,_ (*a* + *c*)_p_, *α*_0s_, *β*_0s,_ *w*_SLDR_) for non-radioresistant SAS and HSC2 cell lines. Figure 4 shows the distribution of the model parameters (*α*_0p*_,*β*_0p*,_ *α*_0s_,*β*_0s_) after the MCMC simulation; (A) SAS cell line, and (B) HSC2 cell line. As expected, the parameters of the progeny cells tended to be lower than those of CSCs. The mean values and standard deviations of the model parameter sets are summarized in Table 1. As shown in Table 1, the weighting factor of the SLDR rate, *w*_SLDR_ is significantly higher than 1.0, indicating that the cell recovery effects of the progeny cells in both SAS-R and HSC2-R cell lines can be enhanced.

**Table 1:** Model parameters for predicting cell surviving fraction of SAS families and HSC2 families.

Model parameter	Type of cell line				Unit
	SAS	SAS-R	HSC2	HSC2-R	
**Progeny cells**					
*α*_0p*_	0.208 ± 0.095	0.197 ± 0.093	0.166 ± 0.160	0.088 ± 0.087	Gy^−1^
*β*_0p*_	0.044 ± 0.012	0.041 ± 0.012	0.168 ± 0.054	0.089 ± 0.036	Gy^−2^
(*a* + *c*)_p*_	1.279 ± 0.687	1.355 ± 0.745	1.499 ± 0.911	2.842 ± 1.856	h^−1^
*w*_SLDR_	1.000*	1.059 ± 0.123	1.000*	1.896 ± 0.453	h^−1^
**Stem-like cells**					
*α*_0s_	0.074 ± 0.098		0.194 ± 0.110		Gy^−1^
*β*_0s_	0.027 ± 0.007		0.019 ± 0.010		Gy^−2^
(*a* + *c*)_H_	1.355 ± 0.745		2.842 ± 1.856		h^−1^
*f*_s_	0.012 ± 0.006	0.083 ± 0.046	0.014 ± 0.004	0.127 ± 0.068	–
**Microdosimetry**					
*γ*	0.954		0.954		Gy

*The *w*_SLDR_ is the ratio of (*a* + *c*)_H_ to (*a* + *c*)_p*_ of non-radioresistant cell lines (SAS, HSC2), so the value of SAS and HSC2 is unity.

**Figure 5:**
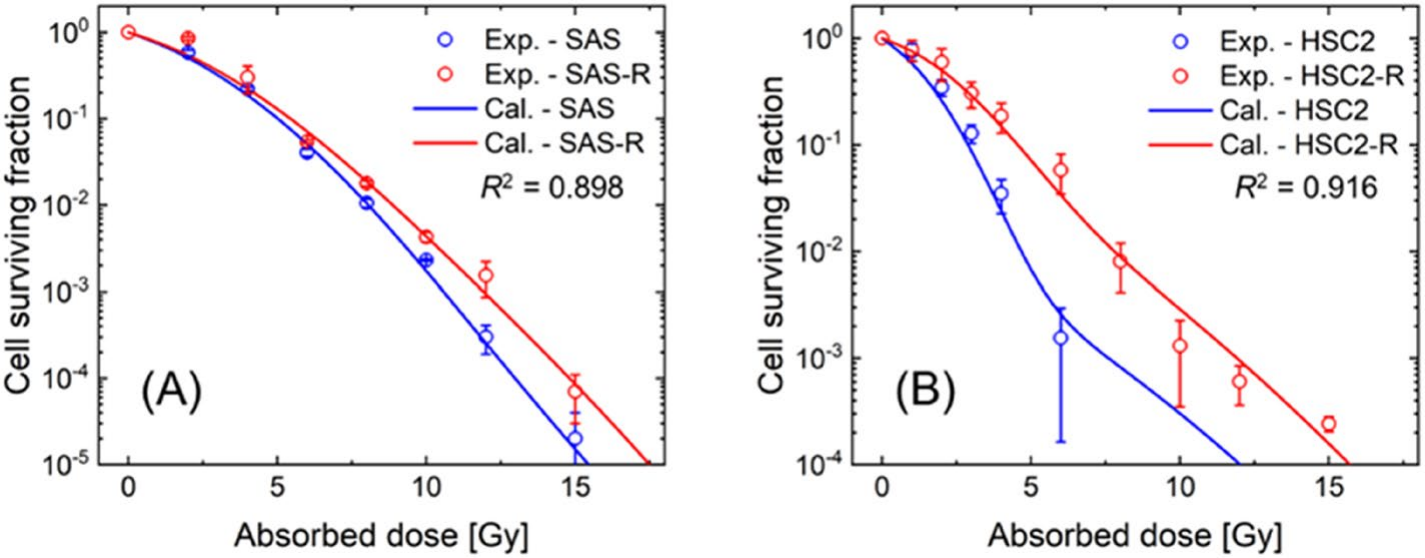
Cell surviving fraction of non-resistant and resistant cell lines after acute irradiation. (**A**) for SAS and SAS-R cell lines, and (**B**) for HSC2 and HSC2-R cell lines. The blue and red circle plots are the mean value of the experimental survival fraction of the non-resistant cells and resistant cells, respectively. The blue and red solid lines are the surviving fraction estimated by the IMK model considering the changes of CSCs fraction and SLDR rate.

Figure 5 shows the comparison of cell survival predicted by the IMK model and the experiments. The accuracy of the model was evaluated by the *R*^2^ value. The results indicated that the IMK model, which considered changes in the CSC fraction and SLDR rate, complied well with the experimental data. Notably, as shown in Fig. 5, the cell survival curves exhibited a sigmoid nature in the relationship between dose and logarithmic survival. The radioresistance exhibited by CSCs against the high dose range (i.e., 10–15 Gy in Fig. 5) showed a similar tendency as our previous report on CSCs^22^. In addition, as shown in Fig. 5, the tendency of the dose response was more pronounced in radioresistant cell lines (SAS-R and HSC2-R) than in non-radioresistant cell lines (SAS and HSC2). These comparisons in Fig. 5 suggest that the changes in both the CSC fractions and DNA repair efficiency (i.e., SLDR) are essential to reproduce the experimental dose–response curves for cell survival.

### Prediction of repair capability during irradiation by the IMK model

**Figure 6:**
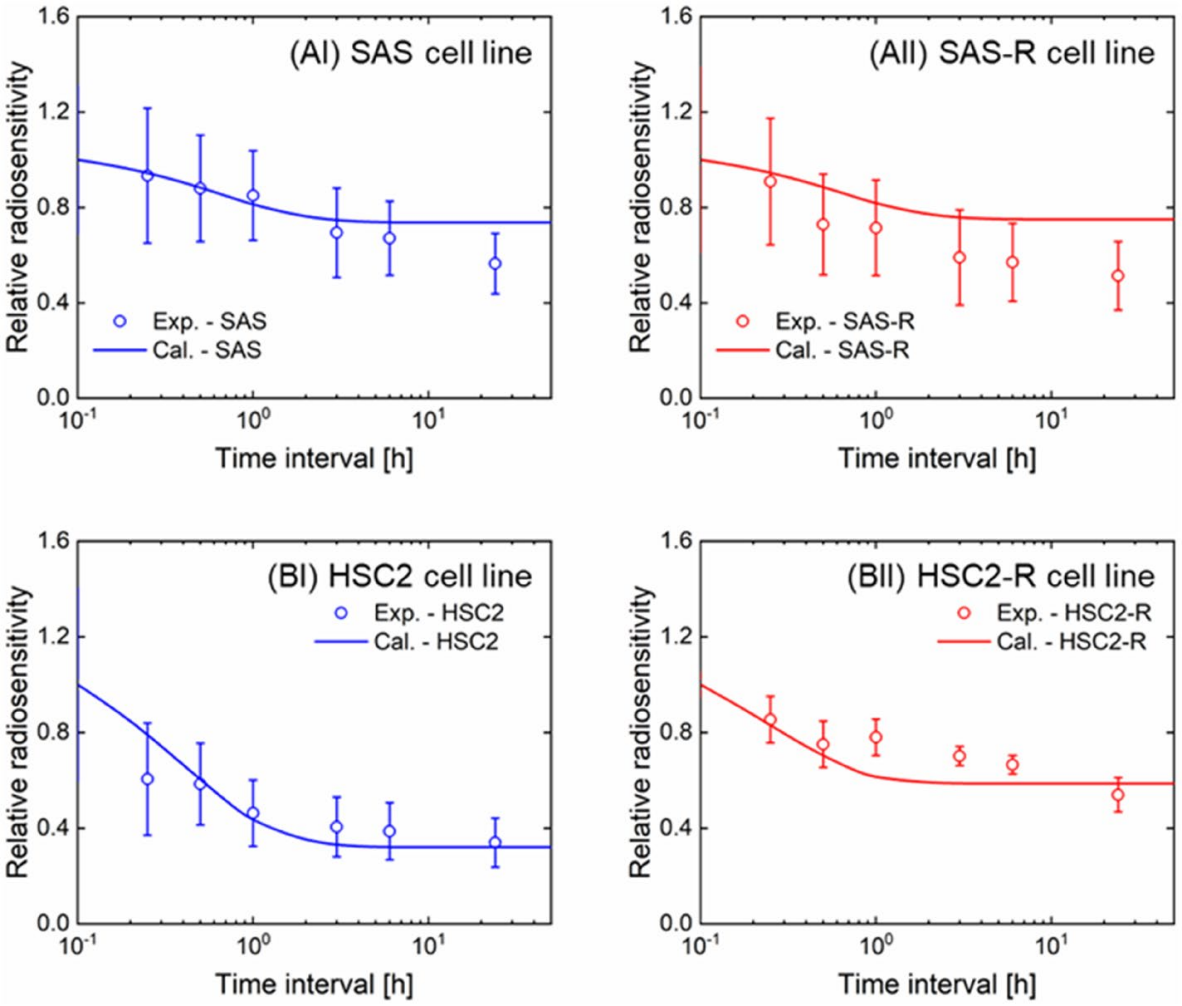
Relative radiosensitivity for split-dose irradiation between the experiment and the model prediction. The circle plot is the experimental cell survival normalized by the cell survival at 4 Gy irradiation (i.e., non-interval irradiation). Solid line is the surviving fraction estimated by the IMK model.

To determine the repair capability of CSCs during fractionated radiotherapy, the cell recovery of the four cell lines in the intervals between irradiation was evaluated. Figure 6 shows the relative radiosensitivity at various time intervals between the irradiations. The relative sensitivity was calculated from the ratio of the surviving fraction after fractionated radiation to that after acute irradiation. As shown in Fig. 5, the relative radiosensitivity estimated by the IMK model (Eqs. (13)–(15)) showed good agreement with the experimental values. As per these comparison results, both the cell survival of non-resistant and resistant cells were saturated at intervals of approximately 3 h, suggesting that the cell recovery during dose fractionation is dominant until a 3 h interval.

**Figure 7:**
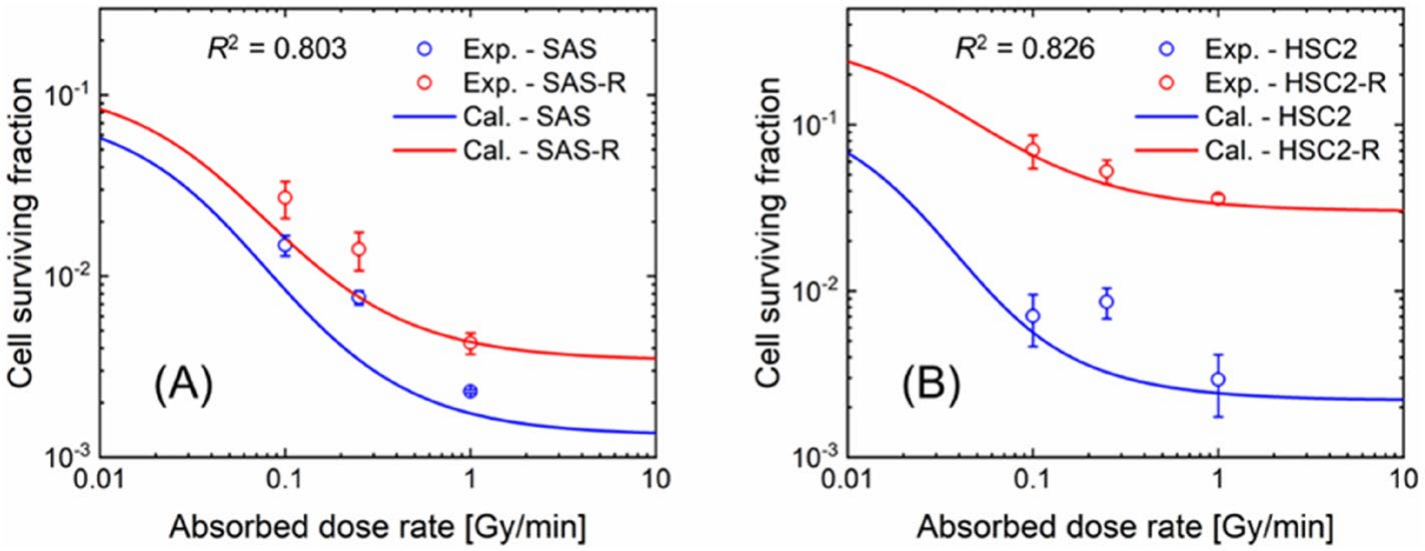
Dose-rate effects measured by experiment and predicted by the IMK model. The logarithmic scale of the relation between dose rate and cell survival. (**A**) is for SAS and SAS-R at a constant dose of 10 Gy, and (**B**) is for HSC and HCS2-R at a constant dose of 6 Gy. Blue and red circle plots are the experimental cell survival of non-resistant cells (SAS and HSC2) and resistant cells (SAS-R and HSC2-R), respectively, while blue and red solid lines are the cell survival of non-resistant (SAS and HSC2) and resistant cells (SAS-R and HSC2-R) estimated by the IMK model.

In addition to the acute irradiation (Fig. 5) and split-dose irradiation (Fig. 6), we further evaluated the dose-rate effects of non-resistant and resistant cell lines. Figure 7 shows the relationship between the absorbed dose rate and surviving fractions of the four cell lines. Dose-rates of 0.1 and 0.25 Gy/min were achieved by multi-fractionated irradiations. As shown in Fig. 7, while the survival of SAS and SAS-R estimated by the IMK model seemed to be slightly lower than the experimental survival, that of the four cell lines was in good agreement with the experimental results, as indicated by the *R*^2^ values. Consistent with the general theory of dose-rate effects that cellular damage leading to cell death is reduced at dose-rates 1.0 Gy/min to 1.0 Gy/h^38^ cell-killing effects were saturated at a dose rate higher than 1.0 Gy/min. In the same manner as the general tendency of dose-rate effects (Fig. 7), the survival results at dose rates below 1.0 Gy/min exhibited significant recovery. The increase in cell survival was also reproduced by the IMK model with high accuracy, suggesting that both the CSC content and the SLDR rate play an important role in predicting the dose-rate effects of non-resistant and resistant cell lines.

## Discussion

The surviving fractions of radioresistant cell lines (SAS-R and HSC2-R) were well reproduced by the IMK model considering the changes in CSC fraction and hypothetical greater cell repair ability (i.e., SLDR) than non-resistant cell lines (SAS and HSC2). DSBs, the potentially lethal lesions caused by irradiation^39^, is mainly repaired by the faster NHEJ pathway rather than the error-free HR pathway^40,41^. Regarding this, it was validated that the cell recovery at 0–3 h between irradiations in Fig. 6 is predominantly attributed to the NHEJ pathway. Although characterization of the radioresistant cells regarding DNA damage repair is still controversial, HR is frequently reported to be associated with radioresistance due to error-free repair^42^. Also, it has been reported that error-prone NHEJ induces genome instability and allows radioresistant clones to expand^43^. In general, NHEJ is the major repair pathway when repair occurs during irradiation in various mammalian cell lines. In addition to enhanced DNA repair capacity, the intracellular reactive oxygen species (ROS) scavenging system seems to be activated in CSCs, leading to protective effects on DNA damage^11^, which may be the major cause of cell death after low-LET irradiation. Further experiments that measure the dynamics of DSBs in ALDH (+) cell populations using the *γ*-H2AX foci formation assay are necessary in the future. In addition, the results presented in this study do not consider the effect of proliferation and cell–cell communication by number of seeded cells in colony formation assay. During fractionated irradiation, cell recovery by proliferation cannot be ignored for clinical daily fractionated irradiations; thus, further model studies and experiments are required to clarify tumor control with high precision.

The dose–response curve predicted by the IMK model showed a linear relationship between the dose and the log of survival after low-dose-rate exposure. This is because, as shown in a previous report^23^, the interaction between two DNA lesions (PLLs) in a site can be ignored because of the damage repair effects, and the secondary term of *β* can be approximated as zero during irradiation. Due to this reason, the cell recovery shown in Fig. 7 seems to be saturated at dose-rate of approximately 0.01 Gy/min. Such drastic cell recovery effects at dose-rate are likely to occur during IMRT. In case of extremely low dose-rate exposure, the inverse dose-rate effects can enhance radiosensitivity; thus, further investigations should be performed when discussing the impact of low-dose-rate exposure on radiosensitivity^44–46^. Meanwhile, in the case of high-dose-rate radiotherapy, which requires a relatively long dose-delivery time, it is necessary to pay attention to the increase in the surviving fraction of radioresistant cells (see the results of radioresistant cell line shown in Fig. 7). Based on these trends, the impact of CSCs and enhanced SLDR (see Table 1) might be crucial in the cell survival. While the surviving fraction of non-resistant and resistant cell lines increased similarly at dose rates below 1 Gy/min, it may be possible that different DNA damage repair mechanisms were induced. Judging from the agreement between the experimental survival and the corresponding model prediction, this model study presents a potential scenario of radioresistant acquisition that changes CSC content and SLDR in progeny cells, which can contribute to the precise understanding of mechanisms contributing to radioresistance. However, experimentally detecting the CSC content is still challenging because the CSC fraction largely depends on the method and markers used. To solve these concerns, in this study, we performed MCMC analysis using mathematical models and experimental survival data to update the percentage of CSC which are attributed to radioresistance in the cell survival curve. For this reason, experimental data for modeling studies are still limited; therefore, further experimental investigations are essential.

Indeed, at 2–6 Gy/min, which is the dose-rate often used for clinical extracorporeal irradiation, dose-rate effects do not seem to be a major problem. However, for multi-field irradiations such as IMRT and SRT, the iso-effective dose-rate decreases owing to the protraction of dose-delivery time^47^. Considering the enhanced cell recovery in SAS-R and HSC2-R (see (a + c) values in Table 1 and Fig. 7), the tumor control probability after a long dose-delivery time, as in the IMRT, might be reduced. Recent clinical studies have reported the effectiveness of shortening the overall treatment time to prevent tumor repopulation and improve local tumor control^48^ due to reoxygenation after radiotherapy and intrinsic radioresistance^49,50^. The results of this study suggest that the inter-fraction time (dose-delivery time) in a treatment should be shortened, because the cell recovery of CSCs might reduce tumor control probability. It should be noted that the present IMK model did not consider the dynamics of the CSC population during irradiation, as well as differentiation and plasticity. Exposure to ionizing radiation induces the epithelial mesenchymal transition (EMT), which is a phenotype closely related to CSC properties and therapy resistance^51^. Zinc finger E-box binding homeobox, one of the core EMT transcription factors, induces cellular plasticity in DNA damage response^52^. This may be derived from the long-term DNA damage response induced as the result of fractionated irradiation. In addition to affecting the tumor cells themselves, irradiation also alters the tumor microenvironment via induction of hypoxia or inflammation. In particular, hypoxia is common in CSC niches^53^. Furthermore, while fractionated irradiation induces the scattering of CSCs^54–57^, clinically relevant radioresistant cells that acquire radioresistance after long-term exposure to fractionated irradiation lose their radioresistance six months after stopping irradiation^58^. From our results in Figs. 4 and 5, the number of cancer stem-like cells increases, which is thought to induce radioresistance in fractionated irradiation. Changes in cancer cell phenotype and microenvironment during fractionated radiation therapy are still unclear, it is necessary to further investigate the characteristics of stem-like cells including the plasticity of CSCs. If the cellular responses of pure CSCs after irradiation could be confirmed, we could further verify the IMK model developed in this study. However, it is very challenging to evaluate the pure radiosensitivity of CSCs (or non-CSCs) because experimentally sorted CSCs differentiate immediately into non-CSCs^59^. To these heterogeneous CSC content, we made a simple assumption that there are two cell population, i.e., progeny cells and stem cells, and verified the model performance (Figs. 5, 6, 7), suggesting that the hypothesis is reasonable. However, for a biological contribution to improve radiotherapy outcomes, the biological characteristics including heterogeneous cell population of CSCs should be incorporated into the IMK model in the future when detailed data are accumulated.

## Conclusions

In this study, we used human oral squamous carcinoma cells, including radioresistant ALDH (+) cells, to investigate not only the mechanisms underlying radioresistance, which is acquired after fractionated irradiation, but also the impact of cell recovery on tumor survival rate. Using a theoretical cell-killing model, termed the IMK model, we successfully interpreted the mechanisms contributing to radioresistance after irradiation, demonstrating that changes in the ALDH (+) fraction and increased SLDR rate are cellular responses in radioresistant cell lines. Cell recovery of radioresistant cell lines is remarkable by both DNA repair and increased CSC content of cancer cells during dose-delivery time. Therefore, the therapeutic effect of IMRT and SRT, which require a relatively long dose-delivery time compared to general radiation therapy, may be reduced. Shortening dose-delivery time should be better for controlling stem-like cells. However, to apply the interpretation by the IMK model to clinical radiotherapy, it is necessary to further estimate the tumor control probability in the future. In addition, the scientific knowledge of experimental data and model studies is still limited, and the underlying mechanisms of stem-like cell responses remain uncertain. Further research is needed to identify the underlying mechanisms; however, this work presents important clues on mechanisms contributing to radioresistance.
